# Diagnostic accuracy of the TrueNat™ MTB plus assay for detecting pulmonary tuberculosis in adults

**DOI:** 10.1371/journal.pone.0327936

**Published:** 2025-12-22

**Authors:** Sarapia P. Mallya, Peter M. Mbelele, Riziki M. Kisonga, Daphine D. Mtunga, Haroun H. Banzi, Stephen S. Mpiima, Muzafaru Twinomujuni, Joel Bazira, Kennedy Kassaza

**Affiliations:** 1 Public Health Laboratory and Disease Surveillance Directorate, Kibong’oto Infectious Diseases Hospital, Siha, Kilimanjaro, Tanzania; 2 Department of Microbiology and Parasitology, Mbarara University of Science and Technology (MUST), Mbarara, Uganda; 3 Department of Microbiology and Immunology, Muhimbili University of Health and Allied Science (MUHAS), Dar es Salaam, Tanzania; 4 National Tuberculosis and Leprosy program, Ministry of Health, Dodoma, Tanzania; 5 Karume Health Centre, Rombo District, Kilimanjaro, Tanzania; Gulu University, UGANDA

## Abstract

Tuberculosis (TB), caused by *Mycobacterium tuberculosis* remains a global threat, affecting 10.8 million people and causes 1.3 million deaths annually. Over 2.5 million cases go undiagnosed partly due to current diagnostic limitations. In particular, smear microscopy is less sensitive, culture is slow and prone to contamination, and the rapid Xpert® MTB/RIF Ultra (Ultra) needs advanced infrastructure. This study assessed the diagnostic accuracy of TrueNat™ MTB Plus assay (TrueNat), a portable, WHO-endorsed point-of-care tool, compared to Ultra, smear microscopy, and Löwenstein–Jensen (LJ) culture. This cross-sectional study enrolled 260 consenting adult participants (≥18 years) with presumptive TB in northern Tanzania. Participants’ sputum samples were tested for *M. tuberculosis* using smear microscopy, LJ culture, Ultra and TrueNat. TrueNat performance was assessed using sensitivity, specificity, predictive values and area under the curve (AUC) against the standard-of-care and a composite reference standard. Age and body-mass-index were summarised using median and interquatile range (IQR). Categorical variables were reported as proportions. Multivariate logistic regression identified TB predictors (p < 0.05). Data analysis and visualization were conducted using R. Among 260 participants, 165 (63.5%) were male, with a median age of 46.0 years (IQR: 35.5–57.3); 15 (5.8%) were HIV-positive, and 52 (20%) had undernutrition. TB was detected in 109 (41.9%) participants by at least one test. TrueNat showed sensitivity/specificity of 34.4%/94.7% vs. smear microscopy, 98.9%/95.3% vs. culture, and 86.2%/95.2% vs. Ultra. The AUC values were 0.75, 0.92, 0.96, and 0.91 compared to smear microscopy, LJ culture, Ultra, and a composite reference method, respectively. Participants reporting weight loss were 2.84 times more likely (95% CI: 1.68–4.84, p < 0.001) to test positive for TB by TrueNat. The TrueNat compared favorably to both Ultra and culture in terms of diagnostic accuracy, offering the added benefits of faster results and greater suitability for resource-limited settings. These findings suggest that TrueNat is a promising test for rapid TB detection in low-resource settings, warranting further studies to evaluate its implementation and impact on patient management.

## Introduction

Tuberculosis (TB), caused by *Mycobacterium tuberculosis* remains a significant public health challenge globally. Over the past decade, annual TB incidence has persistently exceeded 10 million cases worldwide [1]. From 2015 to 2023, the global TB incidence rate declined by only 8.3%, falling far short of the WHO End TB Strategy milestone of a 50% reduction by 2025. In 2023, TB reclaimed its position as the leading cause of death from a single infectious disease, with 1.25 million fatalities, surpassing COVID-19 and causing nearly twice as many deaths as HIV/AIDS [1]. Sub-Saharan Africa accounted for 24% of the 10.8 million global TB cases, underlining the region’s disproportionate burden. Despite the WHO’s ambitious goal to end TB by 2030, progress remains off track. Concerningly, 2.7 million people were undetected and untreated in 2023, amplifying community transmission and increasing mortality by an estimated 50%. In Tanzania, 100,747 cases were notified, leaving a significant diagnostic gap with mortality reduction rate falling below 23%, far short of the 75% target set for 2025 [2].

A critical barrier to meeting these targets is the limited capacity for early diagnosis and the underutilization of diagnostic technologies at the community level. In resource-limited settings, TB diagnosis predominantly relies on sputum smear microscopy, which has a sensitivity of less than 50% in patients with paucibacillary disease, including those with HIV/AIDS. Culture-based methods such as solid Lowenstein-Jensen (LJ) and liquid Mycobacterium Growth Indicator Tube (MGIT) systems are more sensitive and specific than smear microscopy, but they are susceptible to contamination rate of up to 6% and require 42–56 days for results, delaying critical clinical decisions [3].

To overcome these diagnostic challenges, the WHO has endorsed rapid molecular tests. Since 2010, DNA-based assays like the Xpert® MTB/RIF and Xpert® MTB/RIF Ultra (ultra) have become central to TB diagnosis due to their high sensitivity and specificity, delivering results within 24 hours from various sample types. However, these systems require stable electricity and specialized laboratory infrastructure [4]. Similarly, Line Probe Assays (LPAs) such as Genotype® MTBDRplus and MTBDRsl detect *M. tuberculosis* and provide drug-resistance profiles of *M. tuberculosis* against rifampicin, isoniazid, fluoroquinolones within 48 hours but necessitate skilled personnel, multiple workspaces, and uninterrupted power, limiting their use in decentralized settings such as secondary and primary healthcare facilities. As a result, global utilization of rapid molecular testing remains low at 48%, and only 62% of TB cases are bacteriologically confirmed [5]. In Tanzania, only 33% of TB cases notified in 2022 were bacteriologically confirmed, partly reflecting significant limitations in sample referral systems from primary health care facility and community level, and underutilization of rapid nearly point of care rapid molecular diagnostic test such as TrueNat™ MTB plus assay [6].

In 2020, the WHO endorsed TrueNat™, a chip-based, portable, battery-operated molecular diagnostic system designed for use in primary healthcare settings. TrueNat™ MTB Plus (TrueNat) and TrueNat™ MTB-RIF Dx assays offer a decentralized alternative to Xpert®, capable of dual detection of *M. tuberculosis* and rifampicin resistance. This makes TrueNat particularly suitable for resource-constrained settings lacking reliable power supply and laboratory infrastructure [7]. However, sensitivity and specificity vary depending on the location and diagnostic methods used. For example, a systematic and meta-analyses review show TrueNat has a pooled sensitivity of 86% compared to LJ culture and 88% compared to MGIT culture. Its sensitivity and specificity compared to ultra are 92% and 86%, respectively [8]. Regional studies further illustrate these variations. A study in Uganda involving 250 participants at a tertiary hospital, TrueNat demonstrated a sensitivity of 80.5% and specificity of 98.1% compared to Ultra, and 80.8% and 94.1% against LJ/MGIT [9]. In India, a study involving 364 participants found TrueNat had a sensitivity of 90% and specificity of 98.2% for pulmonary TB [10]. Despite the WHO endorsement of TrueNat, evidence supporting its integration into routine clinical care, particularly in decentralized, resource-limited settings, remains scarce. The variability in its diagnostic performance across different contexts and patient characteristics highlights a critical gap. The lack of locally validated data on TrueNat’s accuracy and reliability limits its widespread adoption in countries like Tanzania, where no evidence currently exists on its performance in TB detection. This study aimed to evaluate the diagnostic performance of TrueNat in detecting *M. tuberculosis* from raw sputum samples in Tanzania. Specifically, it assessed the sensitivity, specificity, positive and negative predictive values, and overall accuracy of TrueNat compared to the WHO-endorsed Xpert® MTB/RIF Ultra, smear microscopy, Loweinstein-Jensen culture, and a composite microbiological reference standard.

## Materials and methods

### Study design

This was a prospective cross-sectional diagnostic accuracy study. Data collection was planned and conducted before the TrueNat index test, ultra, smear microscopy and isolation of *M. tuberculosis* in LJ culture media. This design ensured that data were collected in real time, reducing the risk of selection and recall bias and strengthening the validity of the diagnostic performance estimates.

### Participants

#### Eligibility.

This study included male and female adults (≥18 years) presenting with presumptive pulmonary TB. Presumptive TB was defined by the presence of clinical symptoms and signs consistent with TB, typically including cough lasting at least 2 weeks (with or without blood-streaked sputum), chest pain, difficulty breathing, weight loss of at least 3 kg within 3 months, fever, excessive night sweats and/or loss of appetite. Additional risk factors supporting presumptive TB included prior TB diagnosis or treatment, HIV infection, diabetes, undernutrition, smoking, and occupational exposure to mining. Participants were eligible if they were able to provide sputum samples for laboratory testing. Individuals who were already receiving anti-TB treatment or had received treatment within the preceding 7 days were excluded to avoid interference with the performance of the index and reference tests. Participants were consecutively recruited between 20 March 2024 and 30 September 2024 during routine clinic hours, based on predefined eligibility criteria and willingness to participate. The study period spanned both high- and low-TB burden seasons to minimize seasonal variation in case detection.

#### Study Settings.

The study was conducted at the Kibong’oto Infectious Diseases Hospital (KIDH) in Siha District and Karume Health Centre in Rombo District, both in the Kilimanjaro Region of Tanzania. The KIDH is a national superspecialized hospital for the prevention and control of infectious diseases, with a long-standing role in TB care since 1926. In 2009, it was designated as the national center of excellence for the management of drug-resistant TB. Annually, it manages approximately 150 patients with drug-resistant TB and 700 with drug-susceptible TB. The hospital hosts the Biosafety level 3 Public Health Laboratory (KPHL), a zonal referral laboratory serving northern Tanzania (Arusha, Kilimanjaro, Manyara, and Tanga regions) for TB culture testing. The KPHL is equipped with a full range of TB diagnostic platforms, including smear microscopy, solid Löwenstein–Jensen (LJ) culture, liquid MGIT culture, Xpert® MTB/RIF Ultra, line probe assays (Genotype MTBDRplus and MTBDRsl), and next-generation sequencing (Illumina NextSeq 550, Oxford Nanopore). On the other hand, Karume Health Centre is a peripheral health facility in which sputum samples were collected from both outpatients and inpatients for testing using the TrueNat and Xpert® MTB/RIF Ultra. A subset of samples (>2 mL) from Karume Health Centre was transported under cold chain conditions (2–8°C) to KPHL for LJ culture and additional testing [11].

#### Sample Size.

The minimum sample size required was calculated using Cochrane’s formula [12]. A pulmonary TB prevalence of 49% among presumptive TB cases, reported by Mbelele et al. [13], was used as the basis for calculation. Assuming an anticipated average sensitivity of TrueNat, with 95% confidence intervals and 5% significance level, the required sample size was 236. To compensate for an estimated 10% culture contamination rate on LJ media and non-response rate, an additional 14 participants were included, giving a final target of 260 participants, all of whom were enrolled.

#### Ethics.

Written informed consent was obtained from all participants prior to enrolment and any study procedures. Participants diagnosed with TB were started on anti-tuberculosis treatment according to the National TB Treatment Guidelines of Tanzania. Ethical approval was obtained from the Research Ethics Committee of the Mbarara University of Science and Technology (MUST), Uganda (Ref. No. MUST-2023–1255) and the National Institute for Medical Research (NIMR), Tanzania (Ref. No. NIMR/HQ/R.8a/Vol.IX/4568). Administrative permission was also granted by Kibong’oto Infectious Diseases Hospital and Karume Health Centre.

### Test methods

#### Samples collection and processing.

Each study participant provided at least 5mL of one spot sputum sample. The sputum was homogenized and distributed into three portions, one portion for detecting *M. tuberculosis* in Xpert® MTB/RIF ultra, second portion for TrueNat, and the third portion for isolating *M. tuberculosis* in Loweinstein-Jensen culture Media and detection of acid fast bacilli (AFB) in smear microscopy reference standard tests. The portion of sputum sample for culture was processed using the modified Petroff’s method. Briefly, 3mls of sputum was added to 3mls of sodium hydroxide/N-acetyl cysteine (NaOH/NALC)-Na citrate. The mixture was vortexed and left to stand at room temperature for 15 minutes. Thereafter, phosphate buffer was added to a 50 ml mark of falcon tube then mixed by inversion and concentrated by centrifugation at 3000g for 15 minutes. Supernatants were discarded into a container with 5% phenol. Sediments were suspended in Phosphate buffer solution (PBS) before inoculated in the LJ culture media as recommended by the Clinical and Laboratory Standard Institute (CLSI) and GLI guideline for TB culture and smear microscopy [14].

#### Index test: TrueNat™ MTB Plus assay.

The TrueNat™ MTB Plus assay (Molbio Diagnostics, Goa, India) is a chip-based real-time PCR targeting the *IS6110* and *nrdZ* genes of the *M. tuberculosis* complex. For this test, 1.5 mL of sputum was first treated with two drops of liquefaction buffer and incubated at room temperature for five minutes. From the liquefied sample, 0.5 mL was transferred into a lysis buffer bottle, followed by the addition of two more drops of liquefaction buffer. The mixture was securely capped, thoroughly mixed, and incubated at room temperature for three minutes. The entire content was then transferred into the cartridge sample chamber and processed using the Trueprep® Auto device for DNA extraction. The extracted DNA was subsequently loaded onto the TrueNat MTB Plus chip and analyzed on the Truelab® real-time micro-PCR analyzer, which automatically detected the presence or absence of *M. tuberculosis* DNA. Results were reported as *M. tuberculosis* detected, *M. tuberculosis* not detected, or indeterminate. For positive results, bacterial load was semi-quantitatively categorized as high (Ct < 20), medium (20 ≤ Ct < 25), low (25 ≤ Ct < 30), or very low (Ct ≥ 30). Runs were validated by the instrument and reported as either valid or invalid [15]. All index test results were interpreted blinded to the reference standard results.

### Reference standard tests

#### Xpert® MTB/RIF ultra assay.

The Xpert® MTB/RIF Ultra was used as the reference test to provide a comparison with the index test, as both assays target the DNA of the *M. tuberculosis* complex. It was selected because it is a WHO-endorsed, widely used molecular diagnostic with high sensitivity and specificity, making it a reliable standard for evaluating new TB diagnostic assays. The sputum was tested as recommended by the manufacturer of Xpert® MTB/RIF Ultra and previous reports [16,17]. Briefly, raw sputum was diluted with sample reagent (SR) at a ratio of 1:2, vigorously shaken 20 times, and incubated at room temperature for 15 minutes to reduce *M. tuberculosis* viability by 10^6^ fold as recommended by the manufacturer. At least 2 ml of liquefied sputum was loaded into the Xpert® MTB/RIF ultra cartridge and the Xpert® MTB/RIF module to continue with automatic DNA extraction, amplification and detection of *M. tuberculosis*. The *M. tuberculosis* detection is done by amplifying specific sequence of the *rpoB* gene probed with five molecular beacons A, B, C, D and E, each labelled with a unique fluorophore. During detection, the valid maximum quantification cycle (Cq) was 39.0 for Probes A, B and C and 36.0 for Probes D and E. The *M. tuberculosis* is detected when at least two probes result in CT values within the valid range and a delta Cq min of less than 2.0. Depending on the Cq value, the *M. tuberculosis* is semi-quantified into very low, low, medium and high for Cq values of 28.0–40, 20.5–27.9, 18.5–20.4 and 15–18.4, respectively. The trace result was valid if TB positivity was based in IS1081, IS6110 results only. The assay is negative for *M. tuberculosis* when there is only one or no positive probe is detected.

#### Solid Lowenstein Jensen (LJ) culture medium.

The Solid Lowenstein-Jensen (LJ) culture medium was used as a reference method because it is the traditional gold standard for *M. tuberculosis* complex detection, allowing for direct isolation and confirmation of viable bacilli. It was chosen to compare with the index test since culture can detect live bacteria even when molecular tests may yield false positives due to non-viable DNA, providing a complementary measure of diagnostic accuracy. The LJ culture was processed in accordance with the CLSI guideline and existing standard operating procedures  at the Kibong’oto Public Health Laboratory (KPHL). In summary, 200 μl of sputum sediments were inoculated on two slopes of LJ medium containing either glycerol for detecting *M. tuberculosis* or pyruvate for detecting *M. bovis*. For each batch of the sputum sample cultured, a standard laboratory strain MTB H37Rv strain and un-inoculated LJ medium was used as positive and negative quality control of culture, respectively. Inoculated LJ media were incubated at 37°C and observed weekly for up to 8 weeks before declared negative. *M. tuberculosis* colonies were identified and reported according to locally existing and CLSI standard operating procedures.

#### Smear Microscopy.

Smear microscopy was used as a reference method because it is the most widely available and routinely used diagnostic test for TB in many resource-limited settings. Comparing the TrueNat™ MTB Plus assay (index test) with smear microscopy allows assessment of its performance against a rapid, low-cost, and field-applicable method that detects AFB directly from sputum samples in remote areas where Xpert® MTB/RIF ultra and culture are not available or feasible. A slide smear was prepared from 10 µL of sputum sediment and isolates, stained using Auramine stain (Auramine O-Phenol, 1% acid alcohol, 0.1% Methylene blue), and examined for AFB under LED fluorescent microscopy, following manufacturer instructions. Smear results were reported as negative if no AFB was observed in one length; as the exact number if 3–24 AFB were seen; AFB 1+ if 20–199 AFB were seen; AFB 2+ if 5–50 AFB per field; and AFB 3+ if >50 AFB were observed per field.

#### Data management and Statistical Analysis.

Socio-demographic and clinical information (age, sex, occupation, and presenting symptoms) were collected from participants and medical charts using a standardized semi-structured questionnaire. Data were verified for accuracy, entered into Microsoft Excel, and cleaned prior to statistical analysis and visualization in R software (version 4.3.1) (http://www.R-project.org). Specifically, the Microsoft Excel CSV file dataset was imported using the base R function read.csv(),and data tidying, cleaning, and transformation were performed using the dplyr and tidyr packages. The receiver operating characteristic curves (ROC) and the area under the curve (AUC) values were generated using the pROC package, and plots were visualized with ggplot2. Continuous variables such as age, weight, and body mass index (BMI) were reported as median and interquartile range (IQR). Categorical variables were reported as proportions. The diagnostic accuracy of the TrueNat was assessed by calculating sensitivity, specificity, and positive and negative predictive values, and was compared to Xpert® MTB/RIF Ultra, smear microscopy, LJ culture, and a composite microbiological reference standard (cMRS), defined as positive by either culture or Xpert® MTB/RIF Ultra [16]. The cMRS was used to reduce misclassification and improve the accuracy of diagnostic evaluation, as no single reference test is perfect. Culture can miss paucibacillary or treated TB cases, while Xpert® MTB/RIF Ultra may detect non-viable bacilli or fail to identify some infections [18]. Cohen’s Kappa statistics were used to calculate the agreement between the TrueNat and the reference standard tests. The ROC was used to assess the overall diagnostic accuracy of the TrueNat. The ROC provides a comprehensive measure of test performance by plotting sensitivity against 1 – specificity and calculating the AUC compared to standard diagnostic tests. Univariate and multivariable logistic regression models were used to determine factors associated with TrueNat positivity, expressed as odds ratios with 95% confidence intervals. TB culture contamination or non-*Mycobacterium tuberculosis* cases were excluded from the analysis. All analyses considered a p-value of less than 0.05 as statistically significant.

## Results

### Characteristics of study participants

**Fig 1:**
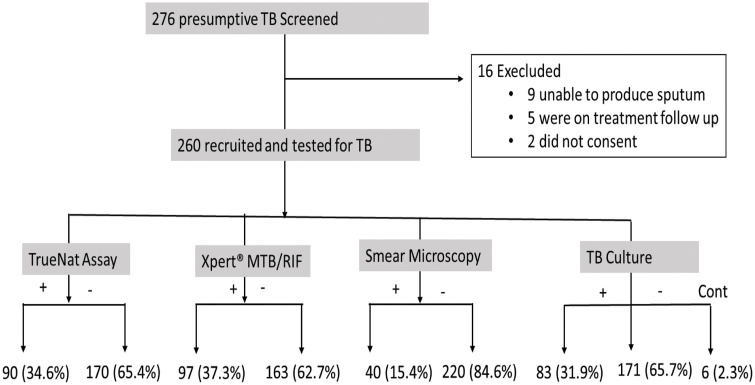
Recruitment and testing procedure. Presumptive tuberculosis (TB) was defined as the presence of symptoms, signs, or risk factors for acquiring TB without bacteriological confirmation. All participants underwent testing with the TrueNat™ MTB Plus assay (index test) and reference standard tests, including Xpert® MTB/RIF Ultra, smear microscopy, and solid Löwenstein-Jensen culture. Test results for all methods were categorized as positive (+), negative (−), or contaminated (cont). Percentages represent the proportion of participants in each category out of the total 260 tested.

Among 276 participants with features of TB, 260 were recruited. The reasons for exclusion in 16 patients are shown in Fig 1. Among the 260 participants, 165 (63.5%) were male, with a median age (IQR) of 46.0 years (35.5–57.3). The socio-demographic and clinical characteristics of the participants are presented in Table 1.

**Table 1:** Socio-demographic and clinical characteristics of study participants.

variable	Category	Frequency	Percentage
Gender	Male	165	63.5
	Female	95	36.5
Age groups	18-30	35	13.5
	31-45	80	30.7
	46-60	93	35.4
	60+	51	20.4
**Median age (IQR), years**	46.0 (35.5–57.3)		
Education Level	Primary	141	54.2
	Secondary	48	18.5
	University	24	9.2
	No formal education	47	17.3
Marital status	Single	68	26.2
	Married/co-habiting	169	65
	Divorced/widow	23	8.8
HIV status	Yes	15	5.8
	No	245	94.2
Nutrition status measured by body mass index (BMI) in Kg/m^2^	Normal (18.5–24.5)	110	42.3
	Mild/moderate (16.5–18.4)	32	12.3
	Severe (<16.5)	20	7.7
	Obese/overweight (≥25)	98	37.7
Median BMI (IQR) Kg/m^2^)	22.9 (19.2-27.0)		
History of TB	Retreatment	17	6.5
	New	243	93.5
History of Smoking status	smokers	57	21.9
	Non-smokers	203	78.1
Alcohol use	Yes	122	46.9
	No	138	53.1
History of working in mining	Yes	24	9.2
	No	236	90.8
Cough	Yes	247	95.0
	No	13	5.0
Chest Pain	Yes	171	65.8
	No	89	34.2
Fever	Yes	206	79.2
	No	54	20.8
Night sweat	Yes	208	80.0
	No	52	20.0

A total of 243 (93.5%) participants had a prior history of TB treatment, and only 15 (5.8%) had HIV co-infection. In total, 57 (21.9%) participants were cigarette smokers. Overall, 52 (20%) participants had undernutrition, defined by a median body mass index (BMI) of less than 18.5 kg//m², while 98 (37.7%) were classified as having overnutrition (overweight), defined by a BMI of more than 25 kg/m².

### Proportion of *M. tuberculosis* complex cases detected

A total of 109 (41.9%) participants had TB detected by at least one of the following tests: culture, Xpert® MTB/RIF Ultra, TrueNat, or smear microscopy. The proportions detected by each test are shown in Fig 1. Xpert® MTB/RIF Ultra identified 97 (37.3%) cases, followed by TrueNat with 90 (34.6%), culture with 83 (31.9%), and smear microscopy with the lowest detection rate at 40 (15.4%). Of 97 TB patients confirmed by Xpert® MTB/RIF Ultra, 5 (5.2%) had trace results. All five patients with trace results tested negative by both TrueNat and smear microscopy, while LJ culture showed two negatives, two positives, and one contamination. Six (2.3%) of 260 participants had contaminated culture results and were excluded from the final analysis comparing TrueNat with LJ culture. Furthermore, of 15 HIV infected individuals, only 4 (26.7%) had TB by either TrueNat or Xpert® MTB/RIF Ultra Assays. LJ culture detected TB in 5 out of 15 HIV-infected individuals and all tested negative by smear microscopy.

### Diagnostic accuracy of TrueNat™ MTB Plus assay

**Fig 2:**
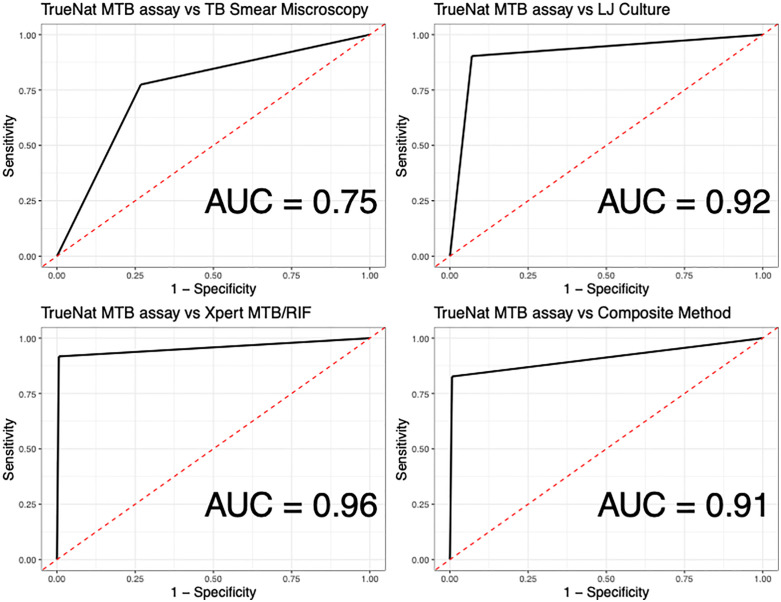
Receiver operating characteristic curves for the TrueNat™ MTB Plus assay. The diagnostic performance of the TrueNat™ MTB Plus assay (index test) was compared with standard reference tests, including Xpert® MTB/RIF Ultra, smear microscopy, and solid Löwenstein–Jensen (LJ) culture, as well as with a composite microbiological reference standard (cMRS), defined as positive by either culture or Xpert® MTB/RIF Ultra. The receiver operator characteristics (ROC) curve plots sensitivity (true positive rate) against 1 − specificity (false positive rate) across different thresholds, providing a visual assessment of index test’s accuracy. The area under the curve (AUC) summarizes overall performance, reflecting the ability of the index test to discriminate between the presence and absence of TB disease. AUC values were interpreted on a scale of 0–1. An AUC of 0.5 indicates no discriminative ability; 0.6–0.7 indicates poor; 0.7–0.8 indicates acceptable/fair; 0.8–0.9 indicates good; 0.9–1.0 indicates excellent; and 1.0 represents perfect discrimination of TB-positive from TB-negative cases by the index test compared with reference standards.

Using smear microscopy, culture, and Xpert® MTB/RIF Ultra as standard reference methods, the sensitivity and specificity of TrueNat in detecting TB were 34.4% and 94.7% compared to smear microscopy; 98.9% and 95.3% compared to Xpert® MTB/RIF Ultra; 86.2% and 95.2% compared to culture (Table 2). The receiver operating characteristic (ROC) curve illustrating the accuracy of TrueNat in detecting *M. tuberculosis*, as defined by the Area Under the Curve (AUC), is shown in Fig 2. The AUC values for TrueNat were 0.75, 0.92, 0.96, and 0.91 when compared to smear microscopy, LJ culture, Xpert® MTB/RIF Ultra, and the composite reference method, respectively (Fig 2). In 220 participants with smear negative results, 59 (26.8%) tested positive by TrueNat. Using Xpert® MTB/RIF ultra and culture as reference standards, TrueNat’s accuracy was similar (Table 2).

**Table 2:** Performance Characteristics of TrueNat™ MTB plus Assay compared to standard of care TB diagnostic tests.

Reference methods		TrueNat™ Assay, n% (95% Confidence interval)							Cohen’s kappa
		Positive	Negative	Total	Sensitivity	Specificity	PPV	NPV	
**Overall accuracy (N = 260)**									
Xpert® MTB/RIF ultra	Positive	89	8	97	98.9 (93.1-99.9)	95.3 (90.6-97.8)	91.8 (83.9-96.1)	99.4 (96.1-100.0)	0.92 (0.88-0.97)
	negative	1	162	163					
	Total	90	170	260					
TB Smear Microscopy	Positive	31	9	40	34.4 (24.9-45.3)	94.7 (89.9-97.4)	77.5 (61.1-88.6)	73.2 (66.7-78.8)	0.34 (0.22-0.45)
	Negative	59	161	220					
	Total	90	170	260					
LJ culture (n = 254)	positive	75	8	83	86.2 (76.7-92.4)	95.2 (90.4-97.8)	90.4 (81.4 - 95.4)	92.9 (87.7 - 96.2)	0.82 (0.75-0.9)
	negative	12	159	171					
	Total	87	167	254					
Composite	Positive	89	19	108	98.9 (93.1- 99.9)	88.8 (82.9 - 93.0)	82.4 (73.6-88.8)	99.3 (95.8-100.0)	0.84 (0.77-0.91)
	Negative	1	151	152					
	Total	90	170	260					
**Accuracy in smear negative (n = 220)**									
Xpert® MTB/RIF ultra	Positive	58	7	65	98.3 (89.7-100.0)	95.7 (90.9-98.1)	89.2 (78.5-95.2)	99.4 (95.9-100.0)	0.91 (0.85-0.97)
	Negative	1	154	155					
	Total	59	161	220					
LJ culture (n = 214)	Positive	45	6	51	80.4 (67.2-89.3)	96.2 (91.5-98.4)	88.2 (75.4-95.1)	93.3 (87.9-96.4)	0.79 (0.69-0.88)
	Negative	11	152	163					
	Total	56	158	214					

Composite means composite microbiological reference Standard (cMRS) defined as a combination of positive and negative results tested with any of Lowenstein-Jensen (LJ) culture, Smear, and Xpert® MTB/RIF Ultra; PPV is positive predictive value and NPV is negative predictive value

### Factors predicting TB detection by TrueNat™ MTB Plus

In a univariate logistic regression model (Table 3), a decrease in participant age (OR: 0.97, 95% CI: 0.95–0.99, p = 0.001) and BMI (OR: 0.92, 95% CI: 0.88–0.97, p = 0.001) was significantly associated with TB detection by TrueNat compared to those with higher age and normal BMI, respectively. Additionally, participants who reported weight loss were 2.84 times more likely (95% CI: 1.68–4.84, p < 0.001) to have TB detected by TrueNat compared to those without weight loss. Cigarette smokers had a 1.66 times higher likelihood (95% CI: 0.09–3.02) of testing positive with TrueNat, though this was only moderately significant compared to non-smokers (Table 3). After adjusting for factors such as sex, HIV status, alcohol consumption, previous TB treatment, presence of symptoms like cough, and education level in a multivariate regression model, age, BMI, and cigarette smoking remained significant predictors of TB detection by the TrueNat (Table 4).

**Table 3:** Univariate logistic regression model for factors predicting TB detection by TrueNat™ MTB Plus Assay.

Predictor variables	Variable category	cOR (95%CI)	p-value
Sex	Female	1 (ref)	0.399
	Male	0.80 (0.47-1.35)	
Age		0.97 (0.95-0.99)	0.001
Marital status	Single	1	
	Married/co-habiting	0.34 (0.64-2.86)	0.439
	Divorced/widow	0.15 (0.30-3.51)	0.932
Weight loss	No	1 (ref)	<0.001
	Yes	2.84 (1.68-4.84)	
BMI **in Kg/M**^**2**^		0.92 (0.88-0.97)	0.001
HIV status	Negative	1 (ref)	0.507
	Positive	0.67 (0.18-2.03)	
Cigarette smoking	Non-smokers	1 (ref)	0.099
	Smokers	1.66 (0.091-3.02)	
Alcohol drinking	No	1 (ref)	0.399
	Yes	0.80 (0.47-1.34)	
History of TB	New	1 (ref)	0.558
	Retreatment	1.35 (0.47-3.64)	
Mining	No	1 (ref)	0.448
	Yes	1.39 (0.58-3.25)	
Cough	No	1 (ref)	0.765
	Yes	1.20 (0.38-4.54)	

BMI is the Body Mass Index, calculated as the ratio of a participant’s weight in kilograms (Kg) to the square of their height in meters (m); cOR is the crude Odds Ratio,

**Table 4:** Multivariate logistic regression for TB detection by TrueNat™ MTB Plus Assay.

Predictor Variables	Variable category	aOR (95%CI)	*P-Value*
Sex	Female	1	0.447
	Male	0.80 (0.45-1.42)	
**Age**		**0.97 (0.94-0.99)**	**0.003**
Education Level	No formal education	1	
	Primary	0.60 (0.28-1.29)	0.185
	Secondary	0.77 (0.30-2.01)	0.599
	University	0.60 (0.18-1.91)	0.389
Marital status	Single	1	
	Married/co-habiting	0.34 (0.64-2.86)	0.439
	Divorced/widow	0.15 (0.30-3.51)	0.932
**BMI in Kg/M** ^**2**^		**0.94 (0.89-0.99)**	**0.024**
HIV status	Negative	1	0.289
	Positive	0.51 (0.13-1.68)	
Cigarette smoking	Non-smokers	1	0.079
	Smokers	2.01 (0.92-4.43)	
Alcohol drinking	No	1	0.388
	Yes	0.80 (0.47-1.34)	
History of TB treatment	New	1	0.903
	Retreatment	1.07 (0.33-3.31)	
Mining	No	1	0.651
	Yes	1.25(0.46-3.23)	

aOR is the adjusted odds ratio computed in this multivariate regression model, BMI is the Body Mass Index, calculated as the ratio of a participant’s weight in kilograms (Kg) to the square of their height in meters (m);

## Discussion

TrueNat™ MTB Plus, a portable point-of-care molecular diagnostic platform, offers significant advantages in sensitivity and specificity. In this study, TrueNat outperformed smear microscopy, especially in patients with low bacterial loads where microscopy is less reliable. Importantly, TrueNat demonstrated diagnostic performance comparable to the Xpert® MTB/RIF Ultra, similar to recent reports in Uganda and Brazil [19,20]. Although Xpert® Ultra provides excellent sensitivity, its use is often limited by infrastructure, cost, and power requirements constraints that are particularly relevant in low-resource settings [21,22]. TrueNat showed almost perfect agreement with Xpert®, with high sensitivity, specificity, and predictive values. Its strong negative predictive value makes it reliable for ruling out TB, while the slightly lower positive predictive value still supports its capacity for true-positive detection.

The sensitivity of TrueNat was relatively lower (<90%) against LJ culture compared to Xpert® MTB/RIF Ultra. This is consistent with a Cochrane review that reported a sensitivity of 87.6% for the TrueNat against TB culture [23]. This may be partly explained by its inability to detect two patients with trace *M. tuberculosis* who were identified by LJ culture in the present study. This argument is consistent with previous reports showing reduced sensitivity of TrueNat in patients with low bacillary load, such as trace, or very low results and smear negative [24,25]. However, compared to Löwenstein-Jensen (LJ) culture, a slow and prone to contamination but sensitive reference method, TrueNat offered the added advantage of rapid turnaround, enabling timely diagnosis and treatment initiation, a critical for reducing TB-related morbidity and mortality. Our findings align with previous data from Uganda by Ssengooba et al., [20]. However, high kappa values and predictive metrics support its clinical utility. Unlike previous studies conducted in well-equipped laboratories at national referral hospitals in Uganda or university TB clinics in Brazil, our evaluation was carried out in primary healthcare settings, where diagnostic tools such as Xpert® and culture are often lacking [20,19]. This highlights the feasibility and potential impact of implementing TrueNat in resource-limited, frontline health facilities. The assay’s rapid and decentralized nature could substantially improve TB detection at the community level. Approximately two in five presumptive cases had TB by at least one method, consistent with earlier reports showing 42% prevalence in similar settings [13,16]. Risk factor analysis revealed that age and undernutrition or weight loss was significantly associated with TB detection by TrueNat, reaffirming the known TB-undernutrition link [26,27]. Cigarette smoking was also associated with a higher risk of TB, as reported in Uganda [20]. Smoking impairs macrophage function and host immunity, facilitating TB progression and increasing the risk of poor treatment outcomes, delayed smear/culture conversion, and loss to follow-up factors critical for TB control efforts [28,29].

Among people living with HIV, detection rates by TrueNat, Xpert® Ultra, and culture were similar, with culture detecting one additional case. Smear microscopy detected none, likely due to the paucibacillary nature of TB in this population. Under ideal conditions, all smear-positive specimens should be detected by TrueNat, given its low limit of detection [30]. In the present study, however, the TrueNat detected more than three-quarters of smear-positive cases and additionally identified 59 smear-negative cases. This resulted in a reduced apparent sensitivity of about one-third when compared with smear microscopy. Similar findings of reduced sensitivity in smear-negative patients have been reported elsewhere [20,24,31], a pattern that our findings confirm given the large smear-negative population tested. Importantly, the TrueNat’s accuracy in patients with smear negative results were higher as in the overall participants. The 9 smear-positive but TrueNat -negative results may partly reflect operational and biological factors, as well as challenges in patient spectrum. For example, some smear-positive specimens may have contained cellular debris or staining artifacts, producing visible acid-fast bacilli on microscopy but insufficient amplifiable DNA for TrueNat. Thus, the observed lower sensitivity is more likely due to the limitations of smear microscopy and operational factors than to an intrinsic weakness of TrueNat.

Compared to a composite microbiological reference method, TrueNat maintained excellent sensitivity and negative predictive value, indicating few missed cases. The close performance of TrueNat to Xpert® Ultra combined with its portability, ease of use, and low infrastructure demands supports its deployment in rural and district-level healthcare settings. Its ability to detect TB in high-risk groups reinforces its role as a valuable diagnostic tool in resource-limited regions.

This study has several strengths. It provides a comprehensive comparison of TrueNat against established diagnostics (Xpert® Ultra, LJ culture, and smear microscopy), enhancing the robustness of findings. Inclusion of high-risk groups such as people living with HIV, those with undernutrition, and smokers adds real-world applicability. Moreover, the study was conducted in health centre and district hospitals with limited access to molecular diagnostics, demonstrating TrueNat’s feasibility in areas with unreliable electricity crucial for settings like Tanzania, where nearly half of TB cases are diagnosed clinically [2]. However, limitations exist. The sample size and restriction to selected facilities in northern Tanzania may limit generalizability. However, the sample size in this study is consistent with previous studies from different settings that reported similar results, supporting the reliability of our findings despite the limited sample [20,19]. Radiological data were not collected, preventing stratification by cavitary disease. Additionally, while LJ culture serves as a diagnostic benchmark, its long turnaround and susceptibility to contamination may underestimate TrueNat’s sensitivity.

## Conclusion

TrueNat demonstrated comparable accuracy to Xpert® MTB/RIF Ultra and Löwenstein–Jensen culture, with the added advantages of faster turnaround time, suitability for resource-limited settings, and the ability to detect 26.8% of participants including those with HIV infection who were missed by smear microscopy. Its superior performance over smear microscopy and its potential for TB detection in participants with low bacterial load such as those with smear negative and HIV infection highlights its value in TB control, especially in peripheral health care facilities like Karume Health Centre in Kilimanjaro region Tanzania and similar settings in sub-Sahara Africa. Future research incorporating liquid culture, diverse geographic settings, large sample size and variables such as cavitary disease and smoking-related outcomes will further clarify its diagnostic role and impact on patient management in real-world conditions

## Supporting information

S1 File A supporting information file containing the Participants’ sociodemographic, clinical, and laboratory metadata has been uploaded as S1 Table. Participants’ metadata. In addition, the Inclusivity in Global Research Questionnaire has been uploaded as S1 Text. Inclusivity in Global Research Questionnaire.
(ZIP)
