# Supplementary material for: Diagnostic accuracy of the TrueNat™ MTB plus assay for detecting pulmonary tuberculosis in adults
Source: PLoS One. 2025 Dec 22;20(12):e0327936. doi: 10.1371/journal.pone.0327936 (PMC12721543; doi:10.1371/journal.pone.0327936)
Supplement: S1 File — A supporting information file containing the Participants’ sociodemographic, clinical, and laboratory metadata has been uploaded as S1 Table. Participants’ metadata. In addition, the Inclusivity in Global Research Questionnaire has been uploaded as S1 Text. Inclusivity in Global Research Questionnaire. (ZIP) [file pone.0327936.s001.zip › S1 File/S1 text. Inclusivity in global research questionnaire.docx]

Inclusivity in global research

PLOS’ policy on inclusivity in global research aims to improve transparency in the reporting of research performed outside of researchers’ own country or community and ensures that PLOS publications reporting global research adhere to high standards for research ethics and authorship. Authors of relevant research articles may be asked to complete the questionnaire below, which outlines ethical, cultural, and scientific considerations specific to inclusivity in global research. This questionnaire may be requested when researchers have travelled to a different country to conduct research, if research uses samples collected in another country, research with Indigenous populations or their lands, or if research is on cultural artefacts. Researchers travelling to another country solely to use laboratory equipment will not normally be required to complete the questionnaire. However, the questionnaire can be requested at the journal’s discretion for any submission – if you have been requested to complete this questionnaire by the PLOS journal you submitted to, please do so.

Please complete the questionnaire below and include this as a Supporting Information file with your manuscript. Note that if your paper is accepted for publication, this checklist will be published with your article in the supporting information files. Please ensure that you reference the checklist in the main body of your manuscript. We suggest adding a subsection ‘Inclusivity in global research’ to your Methods section and adding the following sentence: “Additional information regarding the ethical, cultural, and scientific considerations specific to inclusivity in global research is included in the Supporting Information (SX Checklist)”

The questions have been designed to be applicable to a wide range of study types, and there are subsections for both human subjects research and non-human subjects research. If any of the questions are not relevant to your research please mark them as “N/A” as appropriate.

**Ethical considerations, permits and authorship**

*This section is applicable to all research types.*

Provide details as to who granted permissions and/or consent for the study to take place in the Methods section of your manuscript. This should include the names of **all** ethics boards, governmental organizations, community leaders or other bodies that provided approval for the study. If individuals provided approval refer to these people by their role or title but do not list their name(s).

Reported on page number: **6**

If there were any deviations from the study protocol after approval was obtained please provide details of these changes in the Methods section of your manuscript.
Did this study involve local collaborators that are residents of the country where the research was conducted or members of the community studied? If you do not have any authors from said communities, please provide an explanation for this below.

Reported on page number: **Not applicable**

**Yes, our study involved local collaborators who are residents of the country where the research was conducted. Local co-investigators, research assistants, and clinical staff contributed to recruitment, data collection, and analysis. Their involvement ensured the research was contextually appropriate and aligned with local health priorities in Tanzania**

Everyone listed as an author should meet PLOS’ criteria for authorship and all individuals who meet these criteria should be included in the author byline, rather than the acknowledgements. For further information please see the journal’s Authorship Policy.

**Human subjects research (e.g. health research, medical research, cross-cultural psychology)**

Did you obtain written informed consent from a representative of the local community or region before the research took place? How did you establish who speaks for the community? Details of written informed consent obtained from study participants should be reported separately in the Methods section of your manuscript.

**Yes, written informed consent was obtained from the participating hospital administrative authorieties. The participants were identified through local health authorities, who are recognized as legitimate voices for the community in the health sector. Details of individual participant consent are provided in the Methods section of the manuscript.**

How did members of the local community provide input on the aims of the research investigation, its methodology, and its anticipated outcome(s)?

**The study was motivated by challenges in TB detection in remote areas with limited access to diagnostics. While the local community was not consulted directly, input was provided indirectly through healthcare providers experiencing these diagnostic challenges. The study’s approach was also endorsed by international organizations, such as WHO, despite limited local evidence to support adoption in Tanzania**

When engaging with the local community, how did you ensure that the informed consent documents and other materials could be understood by local stakeholders?

**Although the local community was not engaged directly, informed consent documents and study materials were developed in consultation with local healthcare providers who interact with the community. These materials were written in plain language and translated into Kiswahili languages to ensure clarity and comprehension for participants. Prior to recruitment, participants were given the opportunity to ask questions to the investigators or to a community member or legally authorized representative who was not part of the research team. Lastly, the study was presented to clinical team at the health facility.**

Will the findings of the research be made available in an understandable format to stakeholders in the community where the study was conducted (e.g. via a presentation, summary report, copies of publications, etc.)? Please provide details of how this will be achieved.

**Yes, the findings of the research will continue be shared with local stakeholders in an understandable format. Summaries of the results were provided to healthcare providers in the study area, and key findings were presented at local health meetings. Additionally, lay summaries and copies of publications will be made available to relevant community representatives and local health authorities to facilitate understanding and potential application of the results.**

**Non-human subjects research using specimens/ animals collected as part of the study, or those housed in archival collections. Examples include archaeology, paleontology, botany and zoology.**

Did the permission you obtained from a local authority to perform the study include an agreement on access to outputs and benefit sharing? This may include procedures to enable fair distribution of the benefits and resources arising from the research performed. Please include any details of Prior Informed Consent and Benefit Sharing Agreements obtained. These may be required by field-specific regulations, for example the Convention on Biological Diversity (CBD) and the associated Nagoya Protocol.

**Yes, permission from local authorities included agreements on access to outputs and benefit sharing. Benefits, such as improved diagnostics and capacity building, will be shared with local healthcare providers and institutions. Prior informed consent was obtained from all participants, and the study complied with local regulations; no biological materials were exported**

If the material used in your study was imported, please A) provide the year it was imported and B) indicate whether permits were obtained to import/export the materials used, C) provide details of any permits obtained. If this information is not available, please indicate this.

**No materials were imported. Patient sputum samples were collected and tested immediately according to local procedures**

If you used archival specimens, please state how the material used in your study was acquired by the institute it is held in and provide details of any permits obtained for the original excavations/ sample collection. If this information is not available, please indicate this.

**No archived samples were used in this study; all sputum samples were collected prospectively.**

How was the potential cultural significance of the materials collected in your study to local communities considered in your research design? Were Indigenous peoples and/or local researchers and institutions involved with archaeological excavations / collection of specimens? If so, please provide a description of their involvement.

**The study did not involve culturally significant materials or Indigenous participation. Local researchers and healthcare providers guided sample collection and ensured community-appropriate procedures.**

If your manuscript includes photographs of human remains please indicate whether authors obtained permission from descendants or affiliated cultural communities to do so.

**The manuscript does not include photographs of human remains; therefore, no permission from descendants or affiliated cultural communities was required.**
