# Supplementary figures and images for: Diagnostic accuracy of the TrueNat™ MTB plus assay for detecting pulmonary tuberculosis in adults
Source: PLoS One. 2025 Dec 22;20(12):e0327936. doi: 10.1371/journal.pone.0327936 (PMC12721543; doi:10.1371/journal.pone.0327936)

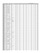

Supplement: S1 File — A supporting information file containing the Participants’ sociodemographic, clinical, and laboratory metadata has been uploaded as S1 Table. Participants’ metadata. In addition, the Inclusivity in Global Research Questionnaire has been uploaded as S1 Text. Inclusivity in Global Research Questionnaire. (ZIP) [file pone.0327936.s001.zip › S1 File/preview-micro.jpg]

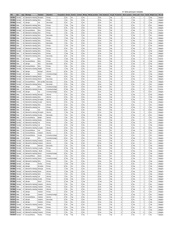

Supplement: S1 File — A supporting information file containing the Participants’ sociodemographic, clinical, and laboratory metadata has been uploaded as S1 Table. Participants’ metadata. In addition, the Inclusivity in Global Research Questionnaire has been uploaded as S1 Text. Inclusivity in Global Research Questionnaire. (ZIP) [file pone.0327936.s001.zip › S1 File/preview-web.jpg]

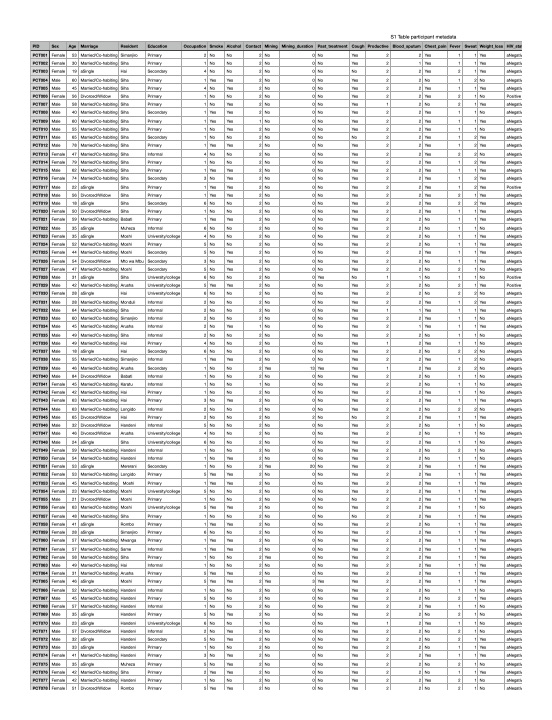

Supplement: S1 File — A supporting information file containing the Participants’ sociodemographic, clinical, and laboratory metadata has been uploaded as S1 Table. Participants’ metadata. In addition, the Inclusivity in Global Research Questionnaire has been uploaded as S1 Text. Inclusivity in Global Research Questionnaire. (ZIP) [file pone.0327936.s001.zip › S1 File/preview.jpg]
